# Do list size and remuneration affect GPs' decisions about how they provide consultations?

**DOI:** 10.1186/1472-6963-9-39

**Published:** 2009-02-26

**Authors:** Michael J van den Berg, Dinny H de Bakker, Gert P Westert, Jouke van der Zee, Peter P Groenewegen

**Affiliations:** 1 NIVEL, Netherlands Institute for Health Services Research, Utrecht, The Netherlands; 2 RIVM, Netherlands Institute for Public Health and the Environment, Bilthoven, The Netherlands; 3 Tilburg University, Faculty of Social and Behavioural Sciences, Tilburg, The Netherlands; 4 Maastricht University, Faculty of Health Sciences, Maastricht, The Netherlands; 5 Utrecht University, Faculty of Social sciences, Utrecht, The Netherlands

## Abstract

**Background:**

Doctors' professional behaviour is influenced by the way they are paid. When GPs are paid per item, i.e., on a fee-for-service basis (FFS), there is a clear relationship between workload and income: more work means more money. In the case of capitation based payment, workload is not directly linked to income since the fees per patient are fixed. In this study list size was considered as an indicator for workload and we investigated how list size and remuneration affect GP decisions about how they provide consultations. The main objectives of this study were to investigate a) how list size is related to consultation length, waiting time to get an appointment, and the likelihood that GPs conduct home visits and b) to what extent the relationships between list size and these three variables are affected by remuneration.

**Methods:**

List size was used because this is an important determinant of objective workload. List size was corrected for number of older patients and patients who lived in deprived areas. We focussed on three dependent variables that we expected to be related to remuneration and list size: consultation length; waiting time to get an appointment; and home visits. Data were derived from the second Dutch National Survey of General Practice (DNSGP-2), carried out between 2000 and 2002. The data were collected using electronic medical records, videotaped consultations and postal surveys. Multilevel regression analyses were performed to assess the hypothesized relationships.

**Results:**

Our results indicate that list size is negatively related to consultation length, especially among GPs with relatively large lists. A correlation between list size and waiting time to get an appointment, and a correlation between list size and the likelihood of a home visit were only found for GPs with small practices. These correlations are modified by the proportion of patients for whom GPs receive capitation fees. Waiting times to get an appointment tend to become shorter with increasing patient lists when there is a larger capitation percentage. The likelihood that GPs will conduct home visit rises with increasing patient lists when the capitation percentage is small.

**Conclusion:**

Remuneration appears to affect GPs' decisions about how they provide consultations, especially among GPs with relatively small patient lists. This role is, however, small compared to other factors such as patient characteristics.

## Background

Time is scarce in general practice. GPs must constantly choose how best to divide their time: between their patients, between patient care and other professional activities, and between their work and their private lives. These decisions are determined, among other variables, by their workload and the number of patients served [1-3]. In systems with fixed patient lists, such as in the Netherlands, list size (the average number of listed patients in a year) corrected for case mix is a good indicator for workload.

GPs' decisions about the provision of care can have important financial consequences, depending on the way in which they are paid. It is commonly assumed that the way in which GPs are remunerated affects their behaviour [4-12]. When GPs are paid per item, i.e., on a fee-for-service basis (FFS), there is a clear relationship between the amount of work and income. More services generate more income. In capitation based systems, this relationship is much weaker, since the annual capitation fee per patient is fixed. In a salaried system, income is not directly related to the patient load.

Previous studies have shown that physicians who were paid under FFS conditions are more likely to have longer working hours, spend more time on patient-related activities, have higher contact rates, more treatments that attract additional remuneration and shorter consultations, and conduct more home visits. Moreover, any form of fund holding or capitation was shown to decrease the total volume of prescriptions written for patients [8,10,13-15].

Most of these studies were international comparisons or consisted of research that described the consequences of changes to the payment system. A problem pertaining to international comparisons is that besides the remuneration systems, there are many other differences between countries that are of influence. Boerma points out that little research has been undertaken on the effects of payment systems because it is difficult to investigate this in a single health care system [15]. The Dutch data we use in this study, however, provide the unique possibility to investigate the relationship between remuneration and list size, because until 2006 Dutch GPs were paid on both a capitation-basis and an FFS-basis, depending on the insurance status of the patient. See appendix 1 for a more detailed clarification of the Dutch payment system [see additional file 1]. In this article, we try to gain more insight into the relationship between remuneration, list size and decisions about how to provide consultations. The main objectives of this study were to investigate a) how list size is related to consultation length, waiting time to get an appointment and the likelihood that GPs do home visits and b) to what extent the correlations between list size and these three variables are affected by remuneration. An important difference vis à vis previous studies is that we investigate this within a single mixed system, which enables us to retain unobserved GP and system wide factors.

### Hypotheses

A high workload can be managed by 'squeezing' or 'spreading' the work. In the first case the time investment remains the same while the GP handles more contacts. This can be done by keeping a close watch on the 'time budget'; avoiding time-consuming encounters such as home visits, and preventing an extension of the consultation length. In the second case, when the work is spread out, the total time investment rises when workload becomes higher.

The first relationship that we investigate is that between the list size and consultation length. Because of the economic advantage of 'squeezing', most GPs will try to avoid going 'overtime' (longer than the booked time slot), during the consultation. However, the greater the capitation share, that is the percentage of publicly insured patients in their practice, the bigger the economic need to keep control of the consultation length. Under capitation conditions, an extra time-investment just generates more work for the same income, whereas under FFS-conditions, there might be more of an incentive to conclude the consultation properly without regarding the time investment. After all, the patient is paying and it is known that patients often find consultations too short [16,17]. So, our first hypothesis (1) is that a large patient list is related to shorter consultations, and (hypothesis 1a) that this relationship will be stronger when the capitation share (proportion of publicly insured) is larger.

Our second point of interest concerns waiting time to get an appointment. Delaying appointments enables GPs to plan and to spread the work better over the week. In an economic sense, delaying appointments for longer is especially attractive under capitation conditions. In addition, some less severe problems will disappear without treatment within a few days so that the amount of work may even fall slightly. Under FFS conditions this is unfavourable; the GP misses some, perhaps relatively simple, consultations and thus income. So (hypothesis 2), we expect that large patient lists are associated with longer waiting time to get an appointment and, (hypothesis 2a) we expect this relationship to be stronger when the capitation part is larger.

The third relationship to be investigated concerns that between list size and home visits. Reducing the number of home visits is profitable under both conditions. Accordingly, we expect a negative relationship between list size and the number of home visits (hypothesis 3). Yet, we expect this relationship to be stronger with a larger capitation share, because under FFS-conditions GPs are (at least partly) compensated for the extra time investment [18].

The above outlined expectations all imply some kind of 'strategic behaviour'. A relatively small list size, however, provides more room for decision-making in this respect. In other words, GPs with a large list simply have no choice but to be economical with time. Previous studies showed that the influence of list size on number of working hours levels off above a certain point [15,19]. Therefore, we expect that the relationship between remuneration on the one hand, and the decisions about how they provide consultations on the other hand, are stronger for GPs with a relatively small weighted list size than for practices with a relatively large weighted list size.

## Methods

### Data

The data we used were derived from the second Dutch National Survey of General Practice (DNSGP-2) [20]. DNSGP-2 was carried out between 2000 and 2002 among 104 general practices in the Netherlands, comprising 195 GPs and accounting for 165.5 GP full-time equivalents. These GPs were compared to a national database of all GPs and they appeared to be representative of the Dutch GP population with respect to age, sex and urbanisation [20]. The GPs were primarily selected on basis on the quality of their electronic medical records. A previous study showed no differences in practice style between GPs participating in a registration network and those who are not [21]. Data were collected using questionnaires, videotaped consultations and routine data collection. The study was carried out in keeping with Dutch legislation on privacy. Compliance with privacy regulations was approved by the Dutch Data Protection Authority. According to Dutch legislation, neither obtaining informed consent nor approval by a medical ethics committee was obligatory for this observational study.

Since the DNSGP-2 contains many different datasets, we will briefly describe the six datasets used. These datasets are also summarised in Table 1; Westert et al. have described the methods and data collection of the DNSGP in greater detail [20].

**Table 1:** Datasets of DNSGP-2, used in this study

**Dataset**	**Variables used**	**Identifiers**	**N**
Videotaped consultations	Consultation length	Unique patient code	1,967 consultations
		Unique GP code	
Postal GP questionnaire	Waiting time to get an appointment	Unique GP code	184 GPs
Recording of type of contact in Electronic Medical Files (six weeks)	Home visit (yes or no)	Unique patient code	67,709 consultations
		Unique GP code	
Practice administration	Insurance status	Unique patient code	399,068 patients
	Sex of patients		
	Age of patients		
	List size		
	Zip code (for selection of deprived areas)		
Patient questionnaire	Self-rated health	Unique patient code	294,999 patients
Database of all participating GPs in DNSGP	Age of GP	Unique GP code	195 (GPs who participated in DNSGP)
	Sex of GP	Unique practice code	
	Practice type		

#### Videotaped consultations

142 of the GPs (73%) in the DNSGP-2 gave permission for the consultations in their surgery to be videotaped. These GPs were likewise representative of the Dutch GP population with respect to age, sex and urbanisation [22]. Of the patients, 88% gave informed consent to participate in the study. Approximately 20 consultations of every GP were recorded. To avoid bias due to the camera, the first five consultations were excluded. In total, 2,095 videotaped consultations were observed afterwards and used for research on communication [22,23]. In this study we will only use the clocked consultation length.

#### GP questionnaire

All GPs received a postal questionnaire covering a range of topics about their work. The response to this questionnaire was 96%, with 184 GPs (94%) answering the questions that we used for the waiting time to get an appointment.

#### Electronic medical records

All participating GPs kept electronic medical records of all contacts. Because the type of contact was not always routinely registered, all GPs were asked to record this aspect during a six-week period for all contacts. The result was a successful data collection of 67,709 contacts for 122 GPs in 83 practices.

#### Practice administration

The practice administration of all participating practices contains a short list of all patient characteristics on the practice list: sex, date of birth, insurance status and postal code. There were almost 400,000 patients in the DNSGP-2.

#### Patient questionnaire

A brief written questionnaire was sent to all listed patients. This included some characteristics which are not registered in the practice administration, such as self-rated health. The response was 76.5%.

#### National database of all GPs

Since 1974, NIVEL has been keeping a national database of all GPs. This database is updated yearly with new graduates. In this database some basic characteristics are collected such as date of birth, sex, graduation year etc. The database contained data of 7,763 GPs, of who 195 participated in the DNSGP-2.

All these files were merged using patient, GP and practice codes as unique identifiers.

### Measures

#### Dependent variables

##### - Length of consultations

This variable was based on the videotaped consultations. The consultation length was measured using a stopwatch, starting at the first verbal expression and stopping after the last verbal expression. Interruptions to the consultation were subtracted from the total consultation length. After listwise deletion, 1,967 consultations were left.

##### - Waiting time to get an appointment

This variable was measured in the GP-questionnaire with the question: 'How long does it take to get an appointment with you?' GPs were asked to give two answers to this question: firstly when the patient calls in the morning, and secondly, when the patient calls in the afternoon. Response categories were: same day; next day; later. Since these answers can be arranged in a logical, hierarchical order, it was possible to create a Guttman-scale [24]. The answers were recoded into a scale from 0, indicating the same day, even when a patient calls in the afternoon, to 3, indicating a later date, even when the patient calls in the morning. Of all GPs, 8% scored 0; 64% scored 1; 24% scored 2; and 4% scored 3. A previous study showed that this scale correlates significantly (R = 0.54) with other aspects of accessibility that patients report from these practices [25]. This concerns mainly regular appointments for office consultations and not the emergency cases. Office consultations include approximately 75% of all contacts [26].

##### -Whether or not patients received a home visit

This is a dichotomous variable. For all contacts between a GP and a patient this variable has either 0, no home visit, or 1, a home visit, as an outcome. For this variable, electronic medical records were used.

#### List size

List size was used as an indicator for workload. List size was computed by averaging the number of patients on the list at the beginning of the year and at the end (based on practice administration). This list size at practice level was divided among the GPs within one practice in proportion to their full-time equivalents (FTE), which was derived from the GP questionnaire. For example, a practice has a mid-time population of 5000, two full-time working GPs (1 FTE) and one GP who works 0.5 FTE, the full-time working GPs have a list size of 2000 and the part-timer, one of 1000. As was mentioned in the introduction, some patients incur a higher care demand than others. List size is especially higher for older patients and in deprived areas. To take these differences into account, we transformed list size into a 'weighted list size'. The weight of a patient was:

1 for patients younger than 65 years and not living in a deprived area,

1.18 for patients older than 65 years and not living in a deprived area,

1.10 for patients younger than 65 years living in a deprived area,

1.28 for patients older than 65 years living in a deprived area.

To compute the weighted list size, the number of patients was multiplied with these weights and added up. These weights are the same as those used for the differentiation in capitation fees [27]. The definition of deprived areas was derived from the literature on the identification of these areas in the U.K., particularly the Jarman-index [28]. The Dutch identification of deprived areas is based on the average income level and unemployment rate [29]. To make the interpretation of coefficients and of the intercept easier, this variable was divided by 1000 and centred around the mean.

#### Independent variables at patient level

The following variables were derived from the practice administration:

- Insurance status, was coded as 0 (privately insured) or 1 (publicly insured)

- Age (years)

- Sex, coded as 0 (male) or 1 (female)

-Self-rated health, (0 = very good to moderate) (1= bad or very bad)

This variable was derived from the patient questionnaire and was originally measured on a scale from 1 (very good) to 5 (very bad); this was recoded as a dichotomous variable. Scores 1 to 3 were recoded as 0, and scores 4 and 5 (bad and very bad) as 1.

#### Independent variables at GP level

-Age and sex of GP

Age and sex of all participating GPs were derived from the national database of GPs.

#### Independent variables at practice level

-Proportion of publicly insured patients

To compute this variable, the insurance status of all listed patients in the practice administration was aggregated to practice level. The proportion indicates the share of the patient population for which GPs receive a capitation payment. This variable was also centred around its mean.

-Degree of urbanisation and practice type

These variables were derived from the national database of GPs and were based on the addresses of the practices.

- Proportion of patients with low self-rated health.

The health status of all patients was asked about in the patient questionnaire and was aggregated to practice level on basis of the valid response.

Means and standard deviations of all variables used are presented in table 2.

**Table 2:** Mean and standard deviation of used variables

	mean	Sd
**Dependent**		
Consultation length (contact level)	9.66	4.64
Waiting time to get an appointment (GP level)	1.18	0.59
Home visit (contact level)	8%	
		
**Independent**		
*Practice level*		
% publicly insured (per practice)^1^	65%	4,00
% self-rated health low (per practice)^1^	18%	4.26
Practice type		
Single-handed	34%	
Dual practice	16%	
Group	50%	
Urbanisation		
Urban	44%	
Suburban	20%	
Rural	36%	
*GP level*		
List size	2017	639
Weighted list size	2080	651
Age	46.08	6.46
Sex (female)	24%	
		
*Patient/contact level*		
Age	43.85	23.52
Sex (female)^2^	60%	
Insurance type patient (1 = public)^2^	73%	
Self-rated health low^1^	20%	

^1 ^All listed patients

^2 ^Since the lowest level concerns contacts, these data only contain individual data of patients that visited their GP during a six-week period. Consequently, there are more women, publicly insured and people with relatively low self-rated health than in the whole population because these categories contact their GP more often.

### Statistical analyses

To explore the relationships between the most important variables, correlations were computed (Pearson's R). Multilevel regression and logistic multilevel regression analyses were carried out to assess the hypothesized relationships. The analyses were carried out with the software package MLwiN.

#### Analysis of consultation length

To analyse consultation length, we used a multilevel model with three levels: contacts (1), GPs (2) and practices (3). No separate patient level was included because more contacts with the same patient rarely occur in the data. This means that level 1 is a contact level as well as a patient level. First, a null-model was estimated. This empty model showed a statistically significant variance at practice level (5% of all variance; p < 0.005) and a significant variation at GP level (5%; p < 0.05). Second, the explanatory variables and the other practice, GP and patient characteristics were added to the model, including two interaction variables: (list size * proportion of patients with public insurance) and (list size * insurance status). These interaction variables are necessary to test hypotheses 1a, 2a and 3a concerning the effect of the proportion of publicly insured on the relationship between list size and the outcome measures. The difference between the two interaction variables is that the first measures an effect of a patient population characteristic, irrespective of whether an individual patient is publicly or privately insured; whereas the second interaction variable measures the effect of the insurance status of a specific patient when this patient contacts the GP.

#### The analysis of waiting time to get an appointment

For this variable, (multilevel) regression models were estimated with the GP as level 1 and practice as level 2. In the first step, a null-model was estimated. This model showed an intraclass-correlation of 50% (p < 0.005).

In the second step, we added the other practice and GP characteristics to the model.

#### Analysis of home visits: Yes or No

Home visit (yes or no) was measured at a contact level and has a dichotomous outcome. Therefore, we estimated a logistic multilevel regression model with four levels: contacts (1); patients (2); GPs (3); and practices (4).

We estimated four logistic models, starting with a null model with random intercept. This intercept could vary between patients, GPs and practices. The model showed a statistically significant variance at patient level, practice level and GP level (all p < 0.005). The major part of the variation appeared to be between patients (92% of level 2, 3 and 4 together). In the second step we added the other variables, including the two interaction terms.

Since in our last hypothesis we stated that the relationship between remuneration on the one hand, and the decisions about how they provide consultations on the other hand, are stronger for GPs with a relatively small weighted list size than for practices with a relatively large weighted list size, we conducted six additional analyses in order to find out whether the coefficients of list size and remuneration differ according to practice list size. This means that the final models for all of the three variables were repeated for GPs with smaller (below median) and larger (above median) weighted lists. All models were also estimated without the interaction variables. Since we are especially interested in the interaction between list size and remuneration, these models are not reported in the tables, but will be discussed in the text where relevant.

## Results

### Correlations

Table 3 shows the correlations between the dependent variables, list size and the proportion of patients in the population for which GPs receive a capitation payment. List size is negatively correlated with consultation length (-0.09). The number of home visits is negatively related to the length of the waiting time to get an appointment, (R = -0.18). Consultation length is weakly but statistically significantly correlated with the proportion of patients for which GPs receive a capitation payment.

**Table 3:** Correlations between dependent variables, list size and % capitation payment (Pearson's R)

		1	2	3	4
1	List size (weighted)				
2	% Capitation payment (publicly insured)	-0.03			
3	Consultation length	-0.09**	-0.06*		
4	Waiting time to get an appointment	-0.08	0.07	0.03	
5	Number of home visits	-0.09	-0.02	-0.13	-0.18*

*p < 0.05; **p < 0.01 (two-tailed)

### Models with explanatory variables

Models with explanatory variables are shown in table 4. In the model for consultation length, the main effect of list size is negative and statistically significant. This coefficient represents the relationship between list size and consultation length subject to the condition that the proportion of publicly insured is average and the patient is privately insured. The interaction of list size and proportion of publicly insured shows no significant coefficient. However, when the interaction variables were left out, the coefficient of list size dropped to -0.74 and was no longer statistically significant. Older people and those with low self-rated health get longer consultations. At practice level, low self-rated health is negatively related to consultation length.

**Table 4:** Regression of remuneration, list size and other practice, GP, and patient characteristics on consultation length, waiting time to get an appointment, and home visit (yes/no) (multilevel regression analysis and logistic regression analysis)

	Consultation length (minutes)	Waiting time to get an appointment (0 through 3)	Home visit yes (1)/no (0)
	B	B	Exp-B
Intercept	11.794	0.904	0.002
**Practice characteristics**			
Proportion of publicly insured (capitation share)	0.034	0.000	1.007
Proportion self-rated health low	-0.192**	0.022	0.994
Urbanization (ref = urban)			
Suburban	-0.970	0.011	1.168
Rural	-1.957**	-0.019	1.405
Practice type (ref = solo)			
Dual	-0.248	0.066	1.097
Group	-0.071	0.289	0.777
**GP characteristics**			
Age	0.020	-0.005	1.002
Sex (female)	0.157	0.002	0.963
Weighted list size	-1.014*	0.065	0,967
Weighted list size * proportion of publicly insured	0.002	-0.004	1.008
**Patient characteristics**			
Insurance status (1 = public)	-0.465		0.999
Age	0.032**		1.061**
Self-rated health low	1.065**		1.570**
Sex (female)	0.327		1.300**
Weighted list size * public insurance	0.400		0.900
			
**Variance components**			
Practice level	0.943	0.176	0.086
Reduction compared to null model	38%	14%	60%
GP level	1.048	0.209	0.102
Reduction compared to null model	0%	0%	32%
Patient level	18.225		2.130
Reduction compared to null model	5%		46%

N	1,967	184	67,709

* p < 0.05; ** p < 0.01

In the model for waiting time to get an appointment, no significant relationships were found.

No significant relationships were found between the likelihood of a home visit and practice, and GP characteristics (including list size). Several patient characteristics, however, show statistically significant coefficients. Women have a higher chance of a home visit than men: exp-b = 1.3; and age and low self-rated health are positively related to the chance of a home visit. Especially the poor self rated health makes a major difference: exp-b = 1.57. The interaction between insurance status and list size is not statistically significant. Leaving out the interaction variables made little difference.

### Smaller and larger practices

Table 5 shows the models 1, 2 and 3 again and the same analyses for GPs with smaller (model 1b, 2b, 3b) and larger practices (model 1c, 2c, 3c). A comparison between smaller and larger practices yielded some remarkable differences.

**Table 5:** Regression of remuneration, list size and other practice, GP, and patient characteristics on consultation length, waiting time to get an appointment, and home visit (yes/no) controlled for practice, GP and patient characteristics.

	Consultation length			Waiting time to get an appointment (0 through 3)			Home visit no (0)/yes (1)		
	1a	1b	1c	2a	2b	2c	3a	3b	3c
	All	Small list	Large list	All	Small list	Large list	All	Small list	Large list
	B	B	B	B	B	B	Exp-B	Exp-B	Exp-B
intercept	11.794	13.597	6.196	0.904	1.789	-0.045	0.002	0.004	0.002
**Practice characteristics**									
Proportion of publicly insured (capitation share)	0.034	0.057	-0.014	0.000	0.012	-0.015	1.007	0.998	1.017*
Proportion of self-rated health low	-0.192**	-0.204*	-0.097	0.022	-0.009	0.066**	0.994	0.978	0.986
Urbanisation (ref = urban)									
Suburban	-0.970	-1.649*	0.242	0.011	-0.096	0.305	1.168	0.655	1.282
Rural	-1.957**	-1.957*	-0.411	-0.019	-0.547*	0.676**	1.405	1.332	1.21
Practice type (ref = solo)									
Dual	-0.248	2.038*	-1.308*	0.066	-0.017	-0.096	1.097	0.723	1.038
Group	-0.071	1.512	-0.338	0.289	0.077	0.141	0.777	0.412**	1.261
**GP-characteristics**									
Age	0.020	-0.034	0.084*	-0.005	-0.003	-0.006	1.002	0.992	1.007
Sex (female)	0.157	-0.651	1.306	0.002	0.053	-0.157	0.963	1.368	0.416**
Weighted list size	-1.014*	-1.346	-2.663*	0.065	-0.046	0.218	0.967	0.842	0.724
Weighted list size * proportion of publicly insured	0.002	0.057	0.036	-0.004	-0.041*	0.012	1.008	0.874**	0.966
**Patient characteristics**									
Insurance status (1 = public)	-0.465	-0.451	-0.451				0.999	1.094	0.949
Age	0.032**	0.031**	0.033**				1.061**	1.066**	1.059**
Self-rated health low	1.065**	1.084**	1.077**				1.570**	1.423**	1.660**
Sex (female)	0.327	0.537	0.132				1.300**	1.302*	1.230**
Weighted list size * public insurance	0.400	1.263	1.283				0.900	1.132	0.975
									
**Variance components**									
Variance practice level	0.943	0.000	1.884	0.176	0.151	0.086	0.086	0.004	0.000
Variance GP level	1.048	1.280	0.000	0.209	0.218	0.207	0.102	0.090	0.116
Variance patient level	18.225	18.982	17.297				2.130	2.390	2.130

N	1,967	996	971	184	92	92	67,709	22,430	45,279

* p < 0.05; ** p < 0.01

Overall, smaller and larger practices (multilevel regression analysis and logistic regression analysis).

In the separate analyses of consultation length, a negative main effect for list size was found in small as well as in large practices, but only in the latter was this coefficient statistically significant. This negative correlation also remained in a model without interaction variables (coefficient of -1.96). No significant interaction effects were found. Furthermore, the variables *practice type *and *urbanisation *show remarkably different coefficients between small and large practices. In the small practices, the consultations appear to be shorter in the suburban and rural areas, which is not the case among the large practices. Among the small practices, dual practices have longer consultations, whereas this coefficient is negative for the larger practices.

**Figure 1:**
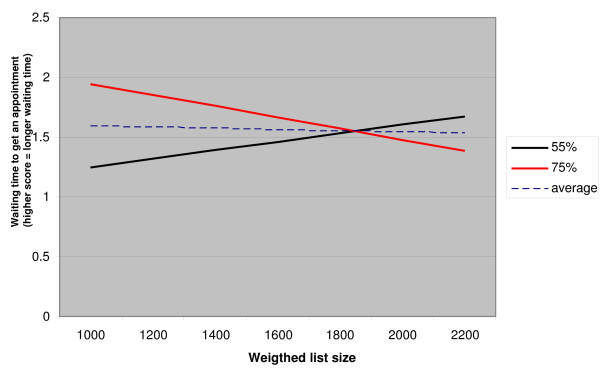
**The relationship between list size and waiting time to get an appointment for small practices with 55% publicly insured patients, small practices with 75% publicly insured patients and small practices with 65% publicly insured patients (average) (male GP in urban group practice, all other variables are average)**.

The models for waiting time to get an appointment also differ between smaller and larger practices. There is a negative effect of the interaction between list size and proportion of patients who are publicly insured among GPs with small practices. To clarify the interpretation, this relationship is displayed in figure 1. The figure shows the correlations between list size and waiting time to get an appointment, for small practices with 55% publicly insured patients (which is 10% below average), and small practices with 75% publicly insured patients, (10% above average) and small practices with an average percentage of publicly insured. The figure refers to male GPs in urban group practices; all other variables were given average scores. Roughly, waiting times get longer with the list size in practices with relatively few publicly insured patients, but shorter in practices with relatively more patients who are publicly insured.

**Figure 2:**
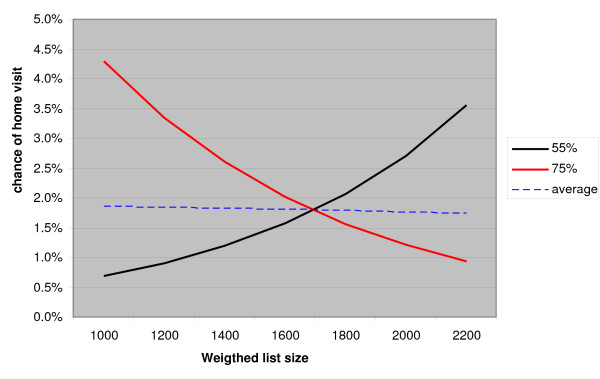
**The relationship between list size and likelihood of a home visit for small practices with 55% publicly insured patients, small practices with 75% publicly insured patients and small practices with 65% publicly insured patients (average) (male GP in urban group practice, female, publicly insured patient with good self-rated health, other variables are average)**.

In the models for home visits (3), we also found a significant interaction between list size and the proportion of publicly insured patients in the small practices (3b). This relationship is displayed in figure 2. It shows that the likelihood of a home visit rises with increasing list size when the proportion of publicly insured patients is relatively small. When this proportion is relatively high, this likelihood decreases with an increasing list size. Yet, it must be noted that the overall chance of a home visit is small in the Netherlands.

Although the sex of the GP has no significant coefficient in the overall model (3a), the models for small and large practices show remarkably different results: a positive odds ratio for female GPs among the small practices and a negative odds ratio among the large practices.

## Discussion and conclusion

The main questions in this article were: a) how is list size related to consultation length, waiting time to get an appointment, and the likelihood that GPs conduct home visits? And b) to what extent are the relationships between list size and these three variables affected by remuneration?

Our results indicate that list size is negatively related to consultation length, especially among GPs with relatively large lists. A correlation between list size and waiting times and list size and likelihood of a home visit was only found for GPs with small practices. These correlations are modified by the proportion of patients for whom GPs receive capitation fees. The associations are, however, relatively weak compared to correlations with patient characteristics such as sex, age and health.

Our theoretical approach led us to assume that, in general, a large patient list would be associated with shorter consultations, longer waiting times and fewer home visits. We expected these correlations for all GPs, because these are ways to manage patient care and maintain control of their workload. We expected these correlations to be stronger when the financial consequences are more favourable. These financial consequences are determined by the share of the population for which GPs receive payment based on FFS. We also expected these interaction effects to be stronger in smaller practices than in larger practices, because small lists provide more room for decision-making.

The consultation length correlates negatively with list size. This finding was in line with hypothesis 1. The relation is, however, weak: a difference of approximately 1 minute per 1000 listed patients. Previous studies also found a negative relationship between consultation length and measures of workload [2,30]. Hypothesis 1a was not confirmed: the relationship between list size and consultation length seems not to be affected by remuneration. Contrary to our expectations, the relationship between list size and consultation length was stronger and only statistically significant among GPs with large practices. In most studies where the relationship between workload and consultation length was investigated, list size was used as workload measure. In these studies, conflicting results have been reported (see for an overview Wilson et al. and Hofman-Okkes [16,31]). Many of these studies showed that consultation length is not, or only weakly, related to list size. It has been shown that some other doctor-related factors affect consultation length positively; these include a positive attitude towards the profession and job satisfaction. Mechanic found that those with a high level of job satisfaction were prepared to 'let the patient talk for half an hour or more' [16,32]. Another interesting finding is that low self-rated health is positively related to consultation length. The proportion of patients with low self-rated health is, however, negatively related to consultation length. This probably illustrates the effect of choices that have to be made with respect to the division of time between patients. Obviously, patients with bad health often require more time, but when there are many of them, there is less time per patient.

The hypothesis (2) that the list size lengthens the waiting time to get an appointment, was not confirmed. The interaction-coefficient that we found is in contrast with our hypothesis (2a). We did find a significant interaction between list size and proportion of publicly insured only among the GPs with small practices. This negative interaction indicates that waiting times tend to become shorter with increasing list size when there is a high proportion of publicly insured patients. This finding is remarkable. After all, longer waiting times seem to be more attractive in the case of publicly insured patients for whom GPs only receive capitation fees.

Home visits are more often carried out with female patients, older patients and patients who are relatively unhealthy. Hypothesis 3 was not confirmed. The finding that home visiting is not related to the list size in our total model is in line with previous findings [33]. In the smaller practices we found a slight negative interaction between list size and the proportion of publicly insured patients. This means that the likelihood of a home visit rises with increasing list size when the proportion of publicly insured is relatively low. This is in line with our hypothesis (3b). Patient characteristics seem to be the most important determinants of home visiting rates. Especially those with a poor self rated health have a substantially higher chance to be visited. Obviously, people with a poor health more often suffer with complaints that restrained them to go to the practice. Calnan and Butler did find a negative relationship between list size and home visiting rates, but did not take population characteristics into account [19].

We hypothesized that the relationship between remuneration on the one hand, and indicators for decisions about how they provide consultations on the other hand, are stronger for GPs with a relatively small list size than for those with a relatively large list size. This is partly confirmed by our findings. In the analyses of waiting times and of home visits we did find a significant interaction among the small practices and not among the large practices. A possible explanation for the finding that remuneration seems only to have a small influence might be that Dutch GPs earn a high income compared to most other countries, and therefore, earning enough income is not much of an issue [34,35]. Another explanation for the absence of the expected relationships is that the influence of their payment is small compared to the other factors that influence GPs' behaviour such as medical assessments and the care for the patients' wellbeing. It is also possible that the workload of most GPs is simply so high that they cannot afford to base their decisions on remuneration factors. After all, beyond a certain limit, all GPs will try to reduce their workload no matter whether the extra work is compensated for or not. Another explanation could be that the effect of factors related to morbidity was insufficiently taken into account. Publicly insured patients are on average less healthy than privately insured patients. We tried to correct for this by controlling for self-rated health but more detailed corrections may be possible.

Some shortcomings of this study include the following. First, the design of the study is not ideal for investigating coping behaviour. Obviously, since the study is cross-sectional, we can only talk about statistical relations, and real causal relations cannot be shown. Yet, theoretically grounded hypotheses that are tested in a cross-sectional study are at least strong indications for causal relations. Furthermore, as in all studies that use routinely collected data, the data can be biased by the recording behaviour of GPs. However, the type of data that we used contains relatively simple data such as consultation type (home visit, office consultation). Moreover, the data collection was intensively controlled by field workers. In our analyses of the likelihood of a home visit, we dichotomised the dependent variable into 1 (home visit) and 0 (office consultation or telephone consultation). Another way to analyze this is to estimate a multinominal model which compares the three types of contacts. This would be an interesting approach for future research. However, we were especially interested in home visits, because we assume that this issue is of greater importance for patients. It is very unlikely that a GP would refuse an office consultation if the patient asks for it, but with regard to the decision to conduct a home visit, GPs are much stricter. A last shortcoming that should be pointed out concerns the waiting time to get an appointment. We asked this in a GP-questionnaire, which means that we only have one measure per GP. Obviously, it would be better to ask patients after every consultation. Yet, as we mentioned earlier, this measure appeared to correlate strongly with other measures for accessibility that were measured on patient level. This indicates that the answers GPs gave were fairly reliable.

## Competing interests

The authors declare that they have no competing interests.

## Authors' contributions

MJB was involved in the original idea, design, analysis and interpretation of the data and wrote the manuscript. DHB was involved in the data collection and the design of the DNSGP, analysis and interpretation of the data and contributed to the critical revision of the manuscript. GPW was involved in the data collection and the design of the DNSGP, the design of the study and contributed to the critical revision of the manuscript. JZ was involved in the data collection and the design of the DNSGP and contributed to the critical revision of the manuscript. PPG was involved in the original idea, design, analysis and interpretation of the data and contributed to the critical revision of the manuscript. All authors read and approved the final manuscript.

## Pre-publication history

The pre-publication history for this paper can be accessed here:



## Supplementary Material

Additional File 1 **Appendix 1 Description of general practice payment system in the Netherlands**. The document provides a short explanation of the general practice payment system in the Netherlands. Click here for file
