# Supplementary material for: Do list size and remuneration affect GPs' decisions about how they provide consultations?
Source: BMC Health Serv Res. 2009 Feb 26;9:39. doi: 10.1186/1472-6963-9-39 (PMC2654894; doi:10.1186/1472-6963-9-39)
Supplement: Additional File 1 — Appendix 1 Description of general practice payment system in the Netherlands. The document provides a short explanation of the general practice payment system in the Netherlands. [file 1472-6963-9-39-S1.doc]

# Appendix 1

## General practice in the Netherlands

- Almost all Dutch citizens were registered with a GP.
- In 2001, there were 6,438 FTE general practitioners working in the Netherlands. In that year the Dutch population was 15,983,103. This represents a GP density of 2,483 in 2001. The most recent numbers show a density of 2,331 [36].
- GPs in the Netherlands have a gatekeeper function. Nearly all medical complaints are first presented to a GP. Consulting secondary care hardly ever happens without a referral from the GP. Approximately 96% of all presented health problems were treated by GPs.
- Around 60% of the population was publicly insured in 2001, the remainder was privately insured. The insurance status depends on income. Above a certain income level, people had to insure themselves privately.
- Since GPs received a capitation payment for publicly insured patients, these patients had to be listed in a practice. The GP receives a fixed amount of money per year for every listed (publicly insured) patient. This amount is slightly higher for elderly (above 65 and for patients living in deprived areas.
- Privately insured patients didn’t have to register with a practice. Yet, practically all privately insured were registered with a GP. For these privately insured patients, GPs were paid on a fee-for-service basis. This fee varies according to the type of contact. It comprises a factor 1 for office-consultations, 0.5 for telephone consultations and 1.5 for home visits. Privately insured patients could opt for a deductible excess in exchange for a lower premium.
- It was possible to change one’s GP. This was, however, easier for privately insured patients than for publicly insured.
- All GPs had a mixed population of publicly as well as privately insured patients. Since insurance type is strongly related to income, the ratio between these two varies across areas. GPs in deprived areas have a vast majority of publicly insured patients on their lists, while those in more wealthy areas have more privately insured patients.
- In 2006, the Dutch health insurance system was changed fundamentally. Today, all Dutch citizens have a mandatory private health insurance. GPs get a capitation fee for all listed patient and fee-for-service on top of that.
